# Upcycling of Agro-Food Chain By-Products to Obtain High-Value-Added Foods

**DOI:** 10.3390/foods11142043

**Published:** 2022-07-11

**Authors:** Graziana Difonzo, Silvia Grassi, Maria Paciulli

**Affiliations:** 1 Department of Soil, Plant and Food Sciences, University of Bari Aldo Moro, Via Amendola 165/A, 70121 Bari, Italy; 2 Department of Food, Environmental and Nutritional Sciences, University of Milan, Via Giovanni Celoria 2, 20133 Milan, Italy; silvia.grassi@unimi.it; 3 Department of Food and Drug, University of Parma, Parco Area delle Scienze 47/A, 43124 Parma, Italy; maria.paciulli@unipr.it

Rising challenges for food innovation and environmental issues have led to an increased interest in bio-economy and more sustainable food production. Besides these transitions, a growing number of consumers are shifting to more sustainable diets, preferring “clean label” items, e.g., minimally processed foods and natural products.

The food industry seems to already have the solution to tackle all these challenges.

Indeed, among the agro-food by-products and waste discarded, most of them possess value-added compounds with high functionality and/or bioactivity. Their use represents a renewable source for originating functional compounds to be used in different fields, such as animal feeding, pharmaceutical and cosmetic industries. Additionally, waste and by-products can be exploited as food ingredients to develop new value-added products, with a benefit for the entire food system.

From one point of view, food by-products are attracting enormous attention amongst researchers as potential raw material for the manufacturing of value-added compounds with high functionality and/or bioactivity. On the other side of the coin, their exploitation is hampered due to high limitations in processing innovations. Indeed, it would be controversial to promote by-product upcycling if the required processes do not fulfill the European Green Deal vision. This gap could be overcome by orienting the upcycling processes to green technologies. Among them, fermentation and biotechnological treatments could be the key players.

Controlled microbial fermentation is a promising strategy to valorize high-volume waste stream. The application of solid-state fermentation by different microorganisms (fungi, yeasts, bacteria) to produce several value-added products was analyzed, focusing on the exploitation of lactic acid bacteria as workhorses for the production of flavoring compounds [1]. The authors focused mainly on lactic acid bacteria for the agri-food waste fermentation to produce molecules with specific sensory attributes, by highlighting the potential of using microbial cultures to obtain “natural” flavoring agents, thus making them more attractive for consumption, with market acceptability.

Marcus et al. [2] reported the recent application of filamentous fungi for the valorization of brewer’s spent grain (BSG) by discussing the biochemical makeup of BSG, the biological mechanisms underlying fungi’s primacy to this application and the current applications of fungi in this realm. A wide range of species have been studied for their ability to use BSG as a substrate including those from the genera Aspergillus, Trichoderma, Neurospora, Candida and Rhizopus, among others. These species have been used to produce a diversity of recoverable enzymes from BSG, including α- and β-amylases, cellulases, hemicellulases, proteases and xylanases. Furthermore, fungi showed the ability to increase the nutrient quality of BSG through the digestion of hemicellulose and the production of proteins, amino acids, antioxidants and vitamins.

Duan, Dai and Zhang [3] discussed the use of a novel feruloyl esterase with high enzymatic activity and temperature stability as a sustainable biological catalysis strategy for ferulic acid (FA) production from de-starched wheat bran. Retrieving FA from by-product is relevant due to its healthy properties, such as delayed progression of fatty liver disease, improved vascular function and anti-diabetic and anti-cancer activities. Thus, it is clear that achieving high-value-added FA production from food by-products by an efficient biological catalysis could be a response to the Sustainable Development Goals. In detail, Duan, Dai and Zhang [3] identified feruloyl esterase from *Bacillus pumilus*, which was then cloned and expressed in *Bacillus subtilis* for the first time. The enzyme exhibited an optimal activity at alkaline pH (9.0) and good thermal stability. Additionally, the study demonstrated the highly specific activity of liberating FA from de-starched wheat bran especially in the presence of commercial xylanase. Even if scientifically relevant, the work did not explore the process scale-up as carried out by Radenkovs et al. [4]. The research group proposed a sustainable and eco-friendly approach to extracting bound FA from cereal bran (rye, wheat and oat) suitable for industrial implementation, using single-step enzymatic hydrolysis (EH) by a mixture of lignocellulose-degrading enzymes. The approach demonstrated up to 369.3 mg 100 g^−1^ FA release from rye bran as well as 255.1 and 33.5 mg 100 g^−1^ of FA for wheat and oat bran, respectively. Furthermore, the authors developed a FA purification phase able to guarantee the absence of residual hydroxycinnamates (HCMs) and carbohydrates, obtaining recovered FA with 94.0% purity. The strength of the research findings relays also on the success of process scale-up, which ensured FA release from rye and wheat bran comparable to the small-scale process.

Valencia et al. [5] proposed a relevant study for enzymatic protein hydrolysis by subtilisin, as a green alternative to chemical hydrolysis, to be applied to salmon frames, leftovers from salmon fillet production. The strength of the work relays on the process feasibility also without water addition and pH control, in the vision of a real green biotechnological treatment, even if these extreme conditions did not lead to the best nitrogen recovery. In any case, by investigating the effect of different levels of water addition, enzyme concentration and pH settings, the work provided a solid framework to decide how to modulate the operating conditions to obtain the desired functional properties for high-value-added protein hydrolysates for human foods.

Different authors explored waste and by-products as a source of prebiotic compounds such as oligosaccharides. In this framework, the fructan profile from the roots of several asparagus varieties grown at different locations and pickled at three vegetative statuses was characterized by Viera-Alcaide et al. [6] in order to valorize these by-products as a fructan source. Fructans were extracted with hot water and fractionated into three pools according to their molecular weight; the fructan content was up to 12.5% on fresh weight basis, depending on the variety and sampling date. The authors found that the relative abundance of the three pools depended on the picking moment, reaching a maximum polymerization degree of 25–30 units. The average MW of the three fractions was similar among varieties with 4.8, 8.4 and 9 sugar units, although fructans up to 30 units were identified. Fuso et al. [7] have summarized different results from the exploitation of hazelnut waste as a source of compounds that are potential precursors of xylooligosaccharides and arabino-xylooligosaccharides ((A)XOS), previously defined as emerging prebiotics which are very beneficial for human health. Moreover, they focused on the main methods that have been developed to optimize (A)XOS extraction and purification steps, but also their structural characterization.

Reintegrating the by-products of the food industry into new food formulations represents the closing of the circle of a design aimed at increasing the economic value and reducing the environmental impact of the supply chains in a perspective of circular economy. In this domain, some studies investigated the potential use of food by-products or their extracts as ingredients or additives for new food formulations.

Szkolnicka, Dmytrów and Mituniewicz-Małek [8] tested the potential use of buttermilk, a by-product of the butter-making process, as a substitute for milk in quark cheese production. The authors tested four kinds of sour buttermilk: two from industrial-level production and two from laboratory-level butter production, differing in the source of buttermilk and kind of starter culture. The study showed that, despite the differences found between the samples throughout 3 weeks of refrigerated storage, buttermilk may be effectively used to produce quark cheese with suitable technological properties and good sensory features. Squeo et al. [9] characterized the oils extracted from durum wheat debranning (DP) and milling (MP) by-products, to be proposed as edible oils after refining. An oil content of around 6 and 5% was found for DP and MP, respectively, both being composed of around 62% of polyunsaturated fatty acids and a rich profile of different phytochemicals. After refining, the mixture of DP/MP (60/40 *w/w*) still showed a high amount of phytosterols and policosanols, while the oxidation products were effectively removed, making this product suitable for human consumption. Ko, Dadmohammadi and Abbaspourrad [10] carried out an extensive review of the scientific literature to discuss the potentiality of pomegranate’s rind and seeds, the residual waste after the juice extraction, as sources of functional compounds for food and cosmetic applications. Pectin, fibers, oil and bioactive compounds extracted from pomegranate by-products have been proposed as natural additives for food formulations to improve both the technological and healthy properties. Moreover, their applications in the field of food packaging have also been discussed, underlying the antioxidant and antimicrobial properties, other than coloring, plasticizing and strengthening film abilities.

Overall, this Special Issue gave an overview of several aspects related to the upcycling vision ranging from technological and biotechnological strategies to reuse agro-food by-products, obtaining functional ingredients for high-value-added food production.

## References

[1] Hadj Saadoun J., Bertani G., Levante A., Vezzosi F., Ricci A., Bernini V., Lazzi C. (2021). Fermentation of agri-food waste: A promising route for the production of aroma compounds. Foods.

[2] Marcus A., Fox G. (2021). Fungal biovalorization of a brewing industry byproduct, brewer’s spent grain: A review. Foods.

[3] Duan X., Dai Y., Zhang T. (2021). Characterization of Feruloyl Esterase from Bacillus pumilus SK52.001 and Its Application in Ferulic Acid Production from De-Starched Wheat Bran. Foods.

[4] Radenkovs V., Juhnevica-Radenkova K., Kviesis J., Lazdina D., Valdovska A., Vallejo F., Lacis G. (2021). Lignocellulose-Degrading Enzymes: A Biotechnology Platform for Ferulic Acid Production from Agro-Industrial Side Streams. Foods.

[5] Valencia P., Valdivia S., Nuñez S., Ovissipour R., Pinto M., Ramirez C., Perez A., Ruz M., Garcia P., Jimenez P. (2021). Assessing the Enzymatic Hydrolysis of Salmon Frame Proteins through Different By-Product/Water Ratios and pH Regimes. Foods.

[6] Viera-Alcaide I., Hamdi A., Guillén-Bejarano R., Rodríguez-Arcos R., Espejo-Calvo J.A., Jiménez-Araujo A. (2022). Asparagus Roots: From an Agricultural By-Product to a Valuable Source of Fructans. Foods.

[7] Fuso A., Risso D., Rosso G., Rosso F., Manini F., Manera I., Caligiani A. (2021). Potential valorization of hazelnut shells through extraction, purification and structural characterization of prebiotic compounds: A critical review. Foods.

[8] Szkolnicka K., Dmytrów I., Mituniewicz-Małek A. (2021). The Characteristics of Quark Cheese Made from Buttermilk during Refrigerated Storage. Foods.

[9] Squeo G., Silletti R., Napoletano G., Greco Miani M., Difonzo G., Pasqualone A., Caponio F. (2022). Characterization and Effect of Refining on the Oil Extracted from Durum Wheat By-Products. Foods.

[10] Ko K., Dadmohammadi Y., Abbaspourrad A. (2021). Nutritional and bioactive components of pomegranate waste used in food and cosmetic applications: A review. Foods.

